# Retraction: β-glucan administration improves growth performance and gut health in New Zealand White and APRI rabbits with different breed responses

**DOI:** 10.1371/journal.pone.0307562

**Published:** 2024-07-17

**Authors:** The PLOS ONE Editors

Following publication of this article [1] concerns were raised regarding Fig 4. Specifically:

- In Fig 4E, there appear to be regions of similarity on the right side of the microscopy panel.
- In Fig 4F, there appear to be regions of similarity on the top left side of the microscopy panel.

The corresponding author stated that the Figs 4E and 4F panels were modified to remove tissue fissure microscopy artifact to enhance the appearance of the panels. The authors provided underlying unadjusted images for all representative panels in Fig 4 and unpublished panels used in the quantification of the results presented in Table 8. Whilst the quantitative data were provided for editorial review, the authors did not clarify whether the data quantification was carried out on the original images or the adjusted images.

During editorial reassessment of the article, PLOS noticed that in contrast to the published Data Availability statement, the individual-level data underlying the published results were not provided with the article. Upon editorial request the authors provided the individual-level data underlying the published results. During editorial review of these data PLOS noted disparities and irregularities in the data underlying multiple figures and tables. The corresponding author stated that these errors were introduced during data entry but that they did not significantly affect statistical analysis.

In the underlying data for Table 3, the values stated for final body weight did not appear to match the published values. The corresponding author stated this was due to an adjustment to the values caused by the analysis of covariance applied to these data. Furthermore, the corresponding author stated that the values for the body weight gain between the 2 breeds may have been erroneously switched, which contradicts one of the findings of the article stating that the New Zealand rabbit breed showed better growth performance.

The *PLOS ONE* Editors contacted an independent member of the *PLOS ONE* Editorial Board to assess the concerns. The Editorial Board member concluded that the issues in the underlying quantitative data and the enhancement of the microscopy data raise concerns regarding overall data management associated with this article.

In light of the concerns affecting multiple figure panels and tables that question the integrity and reliability of these data, the *PLOS ONE* Editors retract this article.

AHEF responded but did not clarify agreement or disagreement with the editorial decision. MMAG, AHAEA, AEN, MSA, and SAM either did not respond directly or could not be reached.
